# Complete mitochondrial genome of the sea star *Archaster typicus* (Asteroidea: Archasteridae)

**DOI:** 10.1080/23802359.2019.1666676

**Published:** 2019-09-20

**Authors:** Zheng Bin Randolph Quek, Jia Jin Marc Chang, Yin Cheong Aden Ip, Danwei Huang

**Affiliations:** a Department of Biological Sciences, National University of Singapore, Singapore, Singapore;; b Tropical Marine Science Institute, National University of Singapore, Singapore, Singapore

**Keywords:** Echinodermata, intertidal, phylogeny, sand star, Valvatida

## Abstract

The complete mitochondrial genome of the widespread and common Indo-Pacific sea star *Archaster typicus* has been sequenced in this study. The mitogenome is 16,230 base pairs (bp) in length, with 13 protein coding genes (PCGs), 22 tRNAs and 2 rRNAs. Gene order of its PCGs and rRNAs matches those of nine other asteroid taxa included for comparison in this study, and it has a similar nucleotide composition of 33.08% A, 26.38% T, 25.53% C and 15.01% G nucleotides. Phylogenetic analyses place *A. typicus* as the sister group to *Acanthaster* spp., consistent with previous inferences.

Distributed across the Indo-Pacific are three sea star species from the family Archasteridae (Sukarno and Jangoux 1977), of which *Archaster typicus* Müller & Troschel, 1840 is the most frequently encountered (Chan et al. 2018). Given its ubiquity, *A. typicus* has become one of the most well-studied sea star species, with numerous studies ranging from the examination of its reproductive biology (Run et al. 1988) to characterization of the metabolites produced (Yang et al. 2011). However, genomic data for *A. typicus* remain limited. Therefore, we here sequenced its mitochondrial genome and performed a phylogenetic analysis along with 12 other echinoderm mitogenomes.

Tube feet were subsampled from one *A. typicus* specimen on 7 December 2017 from the intertidal zone of Cyrene Reef, Singapore (1°15′22.9″N, 103°44′49.0″E). Total genomic DNA was extracted using E.Z.N.A Mollusc DNA Kit (Omega Bio-tek), and subsequently purified using DNA Clean and Concentrator (Zymo Research). Tissue samples and genomic DNA have been deposited in the cryogenic collection of Lee Kong Chian Natural History Museum (catalogue no.: HS0082/333081/333082). Genomic DNA was sheared using BioRuptor Pico (Diagenode) and libraries were prepared using NEBNext Ultra II Library Prep Kit (New England BioLabs). Sequencing was performed in 21.26% of an Illumina MiSeq run (250 × 250 bp).

A total of 3,768,666 raw reads were trimmed using Trimmomatic v0.38 (Bolger et al. 2014) under default settings and assembled using SPAdes v3.12.0 (Bankevich et al. 2012). Assembled mitochondrial contigs were identified using BLASTn (e-value 10^−6^) against two *Acanthaster* (Acanthasteridae) mitogenomes (Yasuda et al. 2006). Matched contigs were assembled into one contiguous sequence using CAP3 (Huang and Madan 1999). Putative circularity of the assembly was verified using circules.py v0.5 (Hahn et al. 2013).

The complete mitogenome of *A. typicus* is 16,230 bp in length, comprising 33.08% A, 26.38% T, 25.53% C and 15.01% G nucleotides (GenBank Accession No. MN052674). MITOS2 (Bernt et al. 2013) (RefSeq 81 Metazoa; Genetic Code 9) annotated 13 protein-coding genes (PCGs), 22 tRNA genes, and 2 rRNA genes. Besides NAD1 which has an initiation codon of GTG (valine), all PCGs have an ATG (methionine) initiation codon. Termination codon for all PCGs is TAA, with cytochrome b being TA(A).

For phylogenetic reconstruction, 12 additional mitogenomes – including nine from Asteroidea and three from Echinoidea outgroups – were reannotated using MITOS2 (Bernt et al. 2013). Annotated PCGs and rRNA sequences were extracted, aligned using MAFFT-L-INS-I v7.271 (Katoh and Standley 2013), and concatenated into a single matrix (14,712 bp). Maximum likelihood phylogeny was inferred using RAxML v8.2.11 (Stamatakis 2014) with 100 random starting trees (GTRGAMA model) and 1000 bootstrap pseudoreplicates. Bayesian analysis was conducted using MrBayes v3.2.6 (Ronquist et al. 2012) (Figure 1). Consistent with the close relationship between Archasteridae and Acanthasteridae recovered by previous analyses (Knott and Wray 2000; Mah and Blake 2012; see also Matsubara et al. 2004), *A. typicus* is sister to *Acanthaster* spp. with maximum node support, and is within the order Valvatida.

**Figure 1.**
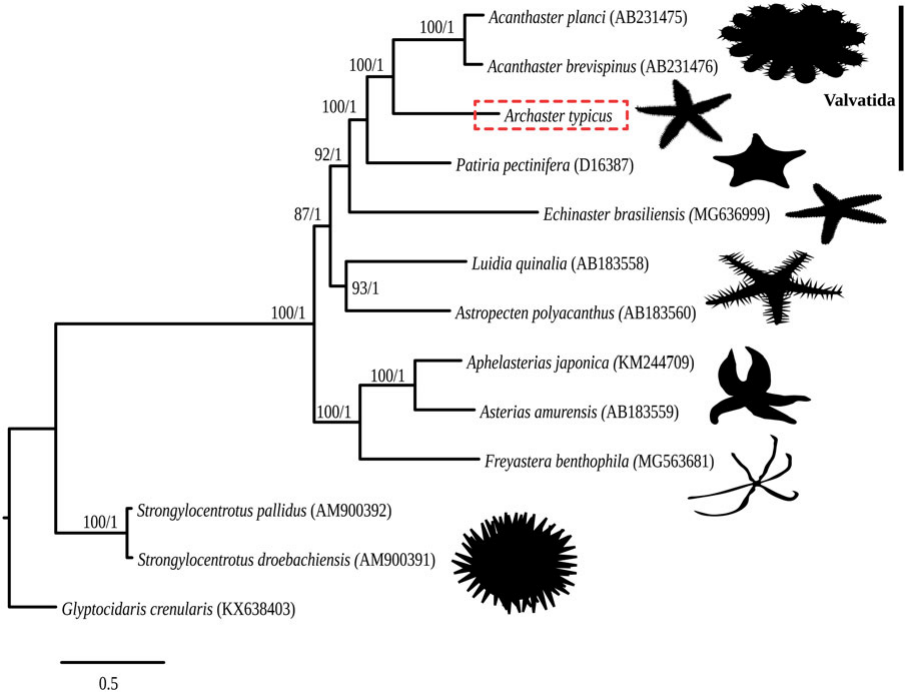
Maximum likelihood phylogeny of Asteroidea with Echinodea as outgroup. Bootstrap support and posterior probability values are shown adjacent to each node.
